# Results from the CLUES study: a cluster randomized trial for the evaluation of cardiovascular guideline implementation in primary care in Spain

**DOI:** 10.1186/s12913-018-2863-x

**Published:** 2018-02-08

**Authors:** Arritxu Etxeberria, Idoia Alcorta, Itziar Pérez, Jose Ignacio Emparanza, Elena Ruiz de Velasco, Maria Teresa Iglesias, Rafael Rotaeche

**Affiliations:** 1 Donostialdea Integrated Healthcare Organization, Osakidetza-Basque Health Service, Biodonostia Health Research Institute, Hernani, Spain; 2 Bidasoa Integrated Healthcare Organization, Osakidetza-Basque Health Service, Irún, Spain; 3 Clinical Epidemiology Unit, Donostia University Hospital, Osakidetza-Basque Health Service, Biodonostia Health Research Institute, CIBERESP, Critical Appraisal Skills Programme, San Sebastian, Spain; 4 Bilbao-Basurto Integrated Healthcare Organization, Osakidetza-Basque Health Service, Bilbao, Spain; 5 Clinical Epidemiology Unit, CIBERESP, Donostia University Hospital, Osakidetza-Basque Health Service, San Sebastian, Spain; 6 Alza Health Centre, Osakidetza-Basque Health Service, Biodonostia Health Research Institute, San Sebastian, Spain; 7 Hernani Health Centre, Aristizabal 1, 20120 Hernani, Spain

**Keywords:** Diabetes, Guidelines, Health plan implementation, Hyperlipidemias, Hypertension, Primary health care, Cardiovascular risk factors

## Abstract

**Background:**

The implementation of evidence-based clinical practice guidelines (CPG) can improve patients care. To date, the impact of implementation strategies has not been evaluated in our context. This study is aimed to evaluate the effectiveness of a multifaceted tailored intervention targeting clinician education for the implementation of three cardiovascular risk-related CPGs (type 2 diabetes, hypertension and dyslipidemia) in primary care at the Basque Health Service compared with usual implementation.

**Methods:**

We conducted a cluster randomized controlled trial in two urban districts with 43 primary care units (PCU). Data from all patients diagnosed with diabetes, hypertension and all those eligible for coronary risk (CR) assessment were included.

In the control group, guidelines were introduced in the usual way (by email, intranet and clinical meetings). In the intervention group, the implementation also included a specific website and workshops.

Primary endpoints were annual HbA1c testing (diabetes), annual general laboratory testing (hypertension) and annual CR assessment (dyslipidemia). Secondary endpoints were process, prescription and clinical endpoints related with guideline recommendations. Analysis was performed at a PCU level weighted by cluster size.

**Results:**

Significant differences between groups were observed in primary outcomes in the dyslipidemia CPG: increased CR assessment for both women and men (weighted mean difference, WMD, 13.58 and 12.91%). No significant differences were observed in diabetes and hypertension CPGs primary outcomes. Regarding secondary endpoints, annual CR assessment was significantly higher in both diabetic and hypertensive patients in the intervention group (WMD 28.16 and 27.55%). Rates of CR assessment before starting new statin treatments also increased (WMD 23.09%), resulting in a lower rate of statin prescribing in low risk women. Diuretic prescribing was higher in the intervention group (WMD 20.59%). Clinical outcomes (HbA1c and blood pressure control) did not differ between groups.

**Conclusions:**

The multifaceted implementation proved to be effective to increase the CR assessment and to improve prescription, but ineffective to improve diabetes and hypertension related outcomes. In order to obtain real improvements when cardiovascular issues are tackled, perhaps other or additional interventions need to be implemented besides education of professionals.

**Trial registration:**

Current Controlled Trials, ISRCTN 88876909 (retrospectively registered on January 13, 2009)

**Electronic supplementary material:**

The online version of this article (10.1186/s12913-018-2863-x) contains supplementary material, which is available to authorized users.

## Background

Hypertension, dyslipidemia and diabetes are the most prevalent clinical conditions in patients with multimorbidity. Current data suggest that there is significant room for improvement in cardiovascular risk management and diabetes care in our setting [1].

The implementation of evidence-based clinical practice guidelines (CPGs) may help to ensure that the care of patients with cardiovascular risk factors or diabetes meets the highest quality criteria and standards. In recent years, we have witnessed the consolidation of a CPG program in both the Basque Country and the Spanish National Health System and this has led to the GuiaSalud Program.

During 2008 and 2009, we saw the publication of the “Clinical practice guideline on the management of lipids as a cardiovascular risk factor” [2], an update to the regional “Clinical practice guideline on hypertension” [3] and the “Clinical practice guideline on type 2 diabetes” [4]. These three guidelines are available from GuiaSalud and have been included in the National Guideline Clearinghouse.

The publication and dissemination of a CPG does not, however, ensure its application in clinical practice. Therefore, effective and feasible implementation plans must be designed, taking into account the organizational context. Multifaceted interventions appear to be effective if they seek to overcome specific barriers [5]. Indeed, tailored interventions seem to be more effective than passive guideline dissemination [6].

According to the Spanish Implementation Manual [7], Grol’s 10-step model [8] provides a suitable theoretical framework for guideline implementation. This model postulates that health professionals pass through different phases or stages (orientation, insight, acceptance, change and maintenance) when achieving a change, with specific barriers predominating in each phase. We undertook a study to explore the barriers to and facilitators of the use of CPGs in primary care using the Delphi technique [9], considering the following domains: presentation, format and usability of CPGs, and internal and external barriers. Among the main barriers identified, we should highlight the lack of any of the following: an executive summary or a brief version of the guideline, an online version, and tools to support both patients and clinicians. The formal presentation of guidelines to clinicians also seems to be very important, and interactive meetings based on real situations conducted by clinical or local leaders are believed to improve guideline acceptance and uptake.

As no single implementation strategy is effective in all contexts, the impact of such strategies needs to be evaluated. The most appropriate design to assess the impact is a randomized controlled trial, with the participating primary care units (PCUs) as the randomization units and measurement of the results at the patient level, that is, a cluster randomized trial [10]. This study is aimed to evaluate the effectiveness of a multifaceted tailored intervention in the implementation of three cardiovascular risk-related CPGs (hypertension, type 2 diabetes and dyslipidemia) in primary care in the Basque Health Service compared with the usual implementation.

## Methods

### Study design, setting, study population

This study is a cluster randomized trial conducted in two urban primary care districts in the Basque Health Service (Ekialde and Bilbao). We assessed the impact of the intervention measuring the change in the outcomes between the year before and the year after the intervention period for both groups (intervention and control) [11], including all patients seeking a medical consultation and that fulfill inclusion criteria. The study methods described here have been reported in detail elsewhere [12].

These districts cover 36.9% of the population of the Basque Country. All 43 PCUs in these two districts were randomized to one of two groups: the intervention group or the control group. All family physicians and nurses who agreed to participate in the study were included. We reviewed electronic medical records from all patients who attended their PCU during the baseline and post intervention periods and met one of the following inclusion criteria:

- A diagnosis of type 2 diabetes documented in their medical record
- A diagnosis of hypertension documented in their medical record
- Eligible for coronary risk (CR) assessment, namely, women 45–74 years of age or men 40–74 years of age with no ischemic heart disease
- Statin treatment started at baseline and post intervention periods
- Coronary heart disease diagnosed during baseline and post intervention periods

Patients were excluded if they were assigned to physicians or nurses who declined to participate in the study, were younger than 14 years of age or did not attend their PCU during the study period.

### Intervention

In the control group, guidelines were introduced in the usual way, that is, they were circulated by email, published on the intranet and presented in clinical meetings in every PCU by local physicians.

In the intervention group, in addition to the aforementioned steps actions, a multifaceted intervention was implemented comprising:

- Presentation meetings led by physicians who took part in the development of each guideline.
- Access to a specifically designed website with very quick and simple access to recommendations application tools such as algorithms linked to recommendations and materials for patients, and links to websites providing drug-related information for professionals.
- Workshops for family physicians and nurses: eight CR workshops for the physicians and a further eight for nursing staff, and four diabetic foot workshops for nursing staff.

The multifaceted intervention was designed taking into account the main barriers and facilitators identified previously [9, 12]. For a detailed description of the intervention see Additional file 1.

### Endpoints

The study endpoints were process and clinical variables, both based on routinely collected clinical information that was obtained for the purpose of this project from electronic medical records. Primary endpoints were process endpoints, while secondary endpoints were a mixture of process and clinical outcomes.

#### Primary endpoints

- Percentage of diabetic patients for whom glycosylated hemoglobin (HbA1c) testing had been performed annually.
- Percentage of hypertensive patients for whom general laboratory tests, including measurement of the albumin-creatinine ratio, had been performed annually.
- Percentage of patients with dyslipidemia between 40 and 74 years of age in men and between 45 and 74 years in women for whom at least one CR assessment had been performed (excluding any patients with cardiovascular disease).

#### Secondary endpoints

- In diabetes, percentage of patients with: HbA1c lower than 7%, blood pressure lower than 140/80 mmHg, annual general laboratory tests, annual CR assessment, foot examinations performed, and percentage of patients newly prescribed metformin treatments out of the total number of patients in which an antidiabetic monotherapy was prescribed for the first time in the same period.
- In hypertension, percentage of patients with: blood pressure lower than 140/90 mmHg (excluding diabetic patients), annual CR assessment, and percentage of patients newly prescribed treatment with diuretics, beta-blockers or angiotensin II receptor blockers (ARBs) out of the total number of patients in which an antihypertensive monotherapy was prescribed for the first time in the same period.
- In dyslipidemia: percentage of patients aged between 35 and 74 years with no cardiovascular disease with a newly prescribed statin treatment with previous CR assessment, percentage of women over 35 years newly prescribed statin treatments with no cardiovascular disease or diabetes, and the percentage of patients with a new diagnosis of coronary heart disease receiving statin treatment.

### Sample size

In our context, annual HbA1c measurements are obtained in a mean of 45–50% of diabetic patients and annual general laboratory test in 35% of hypertensive patients. The research team established by consensus that the intervention strategy would be worthwhile if these mean rates could be increased to 55–60% for diabetes and 43% for hypertension. Assuming an equal standard deviation (SD) of 10 for hypertension, an intracluster correlation coefficient (ICC) of 0.1, and a fixed number of physicians per PCU (*n* = 10), we estimated that about 100 physicians, or 10 PCU, were needed per group to achieve an 80% power (0.20 β error) to detect this difference with an α error of 0.05. We used the independent t-Student test and the correction by the design effect (DE), defined as DE = 1 + (m-1)*ICC, where m = number of physicians/cluster (PCU). Design effect was 2. Thus, the number of clusters (PCU) per group was multiplied by 2, giving a final number of 20 PCU per group (intervention or control) [12].

Close to the end of the follow-up period, a new PCU was created in Bilbao, with eight family physicians, six of them moving from two PCUs previously included in the control group and two new physicians. Patients reallocated to this new PCU were previously assigned to two PCU in the control group, and hence, we decided to pool the data from the new PCU with the rest of control group.

### Randomization and masking

PCUs were allocated by clusters, following a computer-generated randomization sequence. This was performed centrally by a researcher not involved in the study who was blind to the identity of the PCU. Professionals implementing the intervention were not blinded to their group assignment.

### Data collection and analysis

Data extraction from electronic health records was performed centrally by technicians not involved in the study. The intervention was conducted between January 1st and March 15th 2009.

Analysis was performed at the PCU level taking into account the cluster design. Variables were obtained for 12-month periods before and after the intervention and the differences were weighted by cluster size. For WMD, we followed the method proposed by Kerry and Bland [13]. Observed percentages were weighted by the relative size every PCU has in its group (intervention or control). This way we prevented all PCU contributing equally to the group estimate. All analyses were performed using Student’s t-test as implemented in IBM SPSS version 19. Accurate ICCs for primary outcome measures were obtained by linear multilevel modelling using MLxiN v2.21. Analysis was performed on an intention-to-treat basis.

### Ethical considerations

This trial was approved by the Clinical Research Ethics Committee of the Basque Country. The protocol was retrospectively registered in the Current Controlled Trials database, ISRCTN 88876909 (January 13, 2009).

## Results

**Fig. 1:**
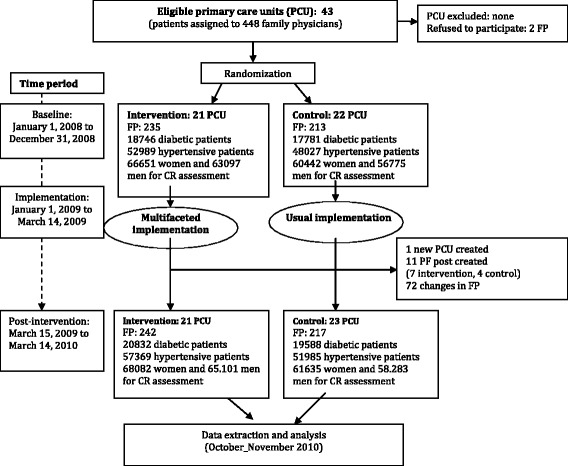
Flow of clusters (PCUs), family physicians and individual participants through each stage of the study

All the PCUs agreed to participate in the study. At an individual level, only two family physicians declined. Data from 43 PCUs and 448 physicians were included in the analysis. Figure 1 shows the number of PCUs and patients involved at each stage of the study.

During the study, 11 new posts were created for physicians, 7 of them in the intervention and 4 in the control group. A total of 72 family physicians out of 448 were changed during the study, the proportion being similar in both groups.

Table 1 summarizes the baseline characteristics of control and intervention PCUs and patients. Table 2 shows the baseline values for outcome variables for diabetes, hypertension and dyslipidemia guidelines in the control and intervention group.

**Table 1:** Baseline characteristics of control and intervention PCUs and patients

	Control group (*n* = 22)	Intervention group (*n* = 21)
PCU at baseline (*n* = 43)		
Number of physicians per PCU	9.68 (4.38)	11.19 (4.26)
Number of diabetic patients per PCU	773 (331)	893 (352)
Number of hypertensive patients per PCU	2088 (780)	2523 (998)
Number of patients per physician	1631 (137)	1665 (128)
Diabetic patients at baseline	(*n* = 17,781)	(*n* = 18,746)
Women (%)	47.76	47.06
Age		
14–39 years (%)	2.33	2.50
40–74 years (%)	59.18	56.00
≥ 75 years (%)	38.49	41.51
HbA1c	7.03 (1.26)	6.99 (1.21)
Hypertensive patients at baseline	(*n* = 48,027)	(*n* = 52,989)
Women (%)	56.23	56.46
Age		
14–39 years (%)	1.22	1.26
40–74 years (%)	57.60	54.89
≥ 75 years (%)	41.175	43.84
Systolic blood pressure	142.77 (17.36)	141.83 (17.11)
Diastolic blood pressure	80.35 (10.22)	80.23 (10.28)
Women eligible for CR assessment at baseline		
Number of women per PCU	2628 (1209)	3174 (1340)
Age	58.91 (8.53)	58.60 (8.78)
Men eligible for CR assessment at baseline		
Number of men per PCU	2468 (1129)	3005 (1223)
Age	55.37 (9.65)	54.56 (9.64)

Values are mean (standard deviation), unless otherwise stated

*CR* cardiovascular risk, *PCU* primary care unit

**Table 2:** Baseline values for outcome variables for diabetes, hypertension and dyslipidemia guidelines in the control and intervention groups

	Control group (% of patients)	Intervention group (% of patients)
Diabetes guideline		
Annual HbA1c testing	77.05	76.18
HbA1c < 7%	44.53	44.94
Annual general laboratory testing	42.13	38.18
Annual coronary risk assessment	10.48	10.88
Annual foot examination	44.87	40.12
Blood pressure < 140/80 mmHg	21.22	21.47
Metformin/All new antidiabetic monotherapy	85.61	85.55
Hypertension guideline		
Annual basic lab examination	24.46	21.81
Blood pressure < 140/90 mmHg	29.98	31.47
Annual coronary risk assessment	10.77	11.46
Diuretics/All new antihypertensive monotherapy	11.41	10.57
Beta blockers/All new antihypertensive monotherapy	6.85	7.77
ARB-II/All new antihypertensive monotherapy	28.75	26.23
Dyslipidemia guideline		
Women with coronary risk assessment	7.73	7.48
Men with coronary risk assessment	7.70	7.06
New statin treatments with previous coronary risk assessment	10.60	10.17
New statin treatments in low-risk women	3.21	3.25
Patients with coronary heart disease receiving statin treatment	76.54	73.56

*ARB-II* angiotensin II receptor blocker

Rates of attendance to the CPG presentation meetings were 69.6 and 62.9% in family physicians and nurses respectively in the intervention group and 66.7 and 62.7% in family physicians and nurses respectively in the control group. Overall, 71.3% of family physicians attended the workshop on CR and diabetes, while 75% of nurses attended the CR workshop and 52.8% the diabetic foot workshop. During the study, there were 25,801 visits to the website, an estimated average of 109 visits per family physician. For a detailed description of the staff attendance at interventions in primary care units see Additional file 2.

Even if the intervention design included feed back on performance data and newsletter reminders, only one newsletter update was sent as a reminder.

Results for the main variables are shown in Table 3. The multifaceted intervention did not result in a higher percentage of diabetes patients having at least one annual HbA1c measurement. No differences were seen in the number of hypertensive patients in whom general annual laboratory tests were performed. On the other hand, there were differences in the percentages of men and women that were screened for CR (weighted mean difference [WMD] 12.91 and 13.58% respectively).

**Table 3:** Primary outcomes related to diabetes, hypertension and dyslipidemia GPCs, expressed as weighted mean differences between intervention and control groups

	WMD	95% CI	*p*	ICC
Annual HbA1c testing (diabetes)	3.83	**−**3.49 to 11.15	0.297	0.0309
Annual general laboratory testing (hypertension)	16.22	−7.35 to 39.80	0.172	0.0039
Eligible women with CR assessment (dyslipidemia)	13.58	5.00 to 22.16	0.003	0.0223
Eligible men with CR assessment (dyslipidemia)	12.91	5.24 to 20.57	0.001	0.0000

*p*-value derived by Student’s t-test

*CR* cardiovascular risk, *CI* Confidence Interval, *ICC* intracluster correlation coefficient, *WMD* weighted mean difference

Table 4 shows the results for the secondary variables related to diabetes, hypertension and dyslipidemia guidelines.

**Table 4:** Secondary outcomes expressed as weighted mean differences between intervention and control groups

	WMD	95% CI	*p*
Diabetes guideline			
HbA1c < 7%	−3.05	−9.16 to 3.07	0.320
Annual general laboratory testing	3.83	−30.74 to 38.41	0.824
Annual coronary risk assessment	28.16	7.22 to 49.08	0.010
Annual foot examination	13.08	−5.30 to 31.45	0.158
Blood pressure < 140/80 mmHg	−2.52	−12.10 to 7.06	0.598
Metformin/All new antidiabetic monotherapy	17.83	−3.22 to 38.88	0.095
Hypertension guideline			
Blood pressure < 140/90 mmHg	3.76	−4.14 to 11.66	0.342
Annual coronary risk assessment	27.55	7.38 to 47.71	0.009
Diuretics/All new antihypertensive monotherapy	20.58	6.44 a 34.73	0.005
Beta blockers/All new antihypertensive monotherapy	−8.24	−21.77 to 5.29	0.226
ARB-II/All new antihypertensive monotherapy	11.32	−10.16 to 32.81	0.293
Dyslipidemia guideline			
New statin treatments with previous coronary risk assessment	23.09	7.26 to 38.92	0.005
New statin treatments in low-risk women	−3.08	−5.20 to −0.94	0.006
Patients with coronary heart disease receiving statin treatment	13.47	−14.36 to 41.30	0.334

*ARB II* angiotensin II receptor blockers, *CI* Confidence Interval, *p p*-value derived by Student’s t-test, *WMD* weighted mean difference

In diabetic patients, rates of CR assessment were significantly higher in the intervention group (WMD 28.16%). There was also a non-significant trend favoring the intervention group for new treatments started with metformin (+ 17.83%). Clinical outcomes (HbA1c and blood pressure) did not differ between the groups.

General laboratory test results improved in both groups, from 42.13 to 55.26% in the control group and 38.18 to 50.83% in the intervention group, and no significant differences were observed between the groups. No differences were observed between groups with respect to foot examinations.

In hypertensive patients, differences in favor of the intervention group were observed in rates of CR assessment (WMD 27.55%) and in the prescribing of diuretics (WMD 20.58%). No significant differences were observed in beta-blocker or ARB prescribing, or in blood pressure control.

With regard to the dyslipidemia guideline, significant differences were also observed in rates of CR assessment before starting new statin treatments (WMD 23.09%). The higher rate in the intervention group translated to a 3.08% lower rate of statin prescribing in women without diabetes or ischemic heart disease. No significant differences were observed in the case of patients with newly diagnosed ischemic heart disease treated with statins.

## Discussion

This study is one of the few wide area and pragmatic cluster randomized trials on the implementation of CPGs conducted in primary care in Spain. Among the observed results it should be highlighted that significant differences between groups were observed in primary outcomes related with dyslipidemia CPG, but the primary endpoints proposed for diabetes and hypertension guidelines did not differ significantly. There are several possible explanations for this uneven effect between guidelines.

On the one hand, it is possible that our intervention, of an educational nature, lead to significant changes in areas where knowledge is still not widespread, such as CR assessment as a tool to decide on whether to initiate treatment with statins. Effective professional education is based on three premises [13]: that the discrepancy between the recommendations and the practice is due to a lack of knowledge, that it is possible to achieve sustained improvements in knowledge and that filling this gap in knowledge can improve care. However, in areas where the real clinical practice is determined by factors related to the attitudes of professionals, therapeutic inertia or lack of coordination among professionals, it is possible that further organizational interventions or interventions in patients are required to increase the chance of success [14, 15].

Ducharme [16] suggest that the key decision in the implementation of a CPG is to choose a simple and actionable message. To implement a limited number of important key recommendations could be more appropriate and effective than trying to implement comprehensive and complicated guidelines. CR assessment was the nexus and the key message for the three guidelines and so was the focus of the intervention. Additionally, a specific new CR assessment tool, validated in Spanish population, was presented and recommended (REGICOR risk score) as a tool to support clinical decisions like statin or antihypertensive prescription. This may explain the greatest impact observed in this variables.

Another possible reason for these findings is the wide margin for improvement given the initial low level of use of CR assessment as a decision tool.

Considering dyslipidemia guideline, the observed increase in the calculation of CR has been accompanied by a modest decrease in the prescribing of new treatments with statins in low-risk women, in contrast to the upward trend of recent years. This result differs from other works in which the improvement of CR assessment was not followed by changes in prescription [17]. In the context of the Mediterranean countries, the unnecessary use of statins is particularly high in low-risk women, where the benefits of treatment are less clear [18]. However, prescribing statins for secondary prevention has not increased significantly, possibly because the baseline was already high with only a small margin for improvement.

There have been few studies on implementation strategies of CR assessment tools and it is unknown which is the best way to encourage their use [19]. In a Dutch study [20], the CR calculation made by clinicians was less effective in improving CR assessment and statin prescribing than the automatic calculation included in the medical record. At the time of this study, in our setting, automatic CR assessment had not yet been incorporated into the electronic health records.

When diabetes guideline is assessed, the lack of improvement observed in primary and clinical outcomes could be explained by various factors: the characteristics of intervention, the pragmatic design of the study or the fact that baseline data were better than expected. The results obtained in the implementation of the guidelines on diabetes are not as good as those found in one previous review [21], but are similar to the findings of recent reviews in this field [14, 22, 23].

Finally, when the main result variable is considered, at the end of the study, HbA1c was measured in three out of four diabetics attending the health center in both intervention and control groups. These figures are higher than expected and can be difficult to improve in a short period of time, as other studies have also found [24–26]. The assessment of the Spanish GEDAPS group on diabetes indicators in the period 1996–2007 shows an improvement in rates of annual HbA1c testing from 59 to 81.6%; however, from 2002 to 2007, the improvement was only 0.6% in absolute terms [27].

In our study, the mean baseline HbA1c level was 7.0%, and did not improve after the intervention, which is in accordance with other studies [24–26, 28]. It is also necessary to take into account the changes in the HbA1c target in the light of the latest evidence showing that tighter control is not accompanied by a decrease in cardiovascular morbidity and mortality [29], leading the recommendation to be less strict in the targets for elderly people with comorbidities.

De Belvis concluded that guidelines and other tools for implementing evidence-based medicine can improve the process of care in diabetes, but is less likely to improve clinical outcomes [22]. Along the same lines, Seitz [14] argues that interventions for clinicians seem to improve clinical outcomes when combined with organizational or interventions for patients. According to Tricco, interventions to improve quality in diabetes can modestly improve HbA1c levels, but this improvement is most marked in patients whose baseline HbA1c is higher than 8.0% [23].

Hypertension guideline didn’t show any improvement in the main variables, as it was observed in the diabetes one.

The effect on blood pressure control was poor, similar to the effect on HbA1c. The percentages of patients with adequate blood pressure control are similar to those found in other research in our country [30], with only 18% of patients with hypertension grade 2 or 3. Improving blood pressure seems difficult to achieve with only educational interventions for health professionals. A Cochrane review [31] focused on interventions to improve control of blood pressure in patients with hypertension showed that education alone, either of patients or clinicians, does not appear to be associated with large net reductions in blood pressure. On the other hand, an organized system of regular review allied to vigorous antihypertensive drug therapy was shown to reduce blood pressure.

Our intervention had the desired effect on diuretic prescribing (associated with a higher rate) and may improve beta-blocker prescribing (associated with a non-significantly lower rate), but not on ARBs. The prescribing of ARBs in our country has followed a strongly upward trend since they came on the market, with a significant influence from specialized care [32]. This improvement in prescribing antihypertensive drugs has also been observed in other clinical trials with interventions similar to ours [33, 34].

The increase in CR assessment in hypertensive patients observed in our study is consistent with previously cited results for diabetes, where changes in blood pressure control were not observed. [35].

Taking in account these data it may be stated that the effectiveness of a strategy to reinforce the implementation of clinical guidelines shows modest and variable results, as suggested in recently published works. According to a systematic review [36], educational interventions can produce statistically significant improvements in cardiovascular guideline adherence compared to usual care, suggesting that they can be more effective than passive guideline dissemination strategies. Another review [15] focused on the implementation of cardiovascular guidelines in primary care showed that the greatest benefits were due to organizational changes, followed by patient education, provider education and provider reminder systems.

The discrepancy observed between the positive effect on CR assessment and the lack of effectiveness on clinical outcomes (HbA1c and blood pressure) is worth some reflection [37]. The concept of CR should be integrated in a shared patient-professional decision making process in order to produce a significant impact in clinical results. Nevertheless, the most adequate strategy to achieve it is still to be agreed [35].

Borgermans [38] proposes that the interventions to improve quality are based on process changes. Also, it should be taken into account that process variables are faster and easier to shown changes than clinic ones [39]. In fact, for this study process variables were defined as primary outcomes. Nevertheless we find disappointing not having observed the expected impact in clinical aspects associated to the implementation of clinical guidelines, being these the main goal of their application in clinical practice.

One limitation of our study is the use of data obtained from the electronic medical records. However, this limitation was already considered in the design of the study. In order to avoid the effect of variability in registration laboratory variables were selected because they are automatically entered into the medical record. As secondary variables, we selected routinely collected data and relevant clinical measures on which physicians rely to optimize patients’ care.

It is possible that 1 year is an insufficient period of time to observe substantial changes at the population level, since implementation is very gradual, as patients attend the health centers.

Small organizational changes could have been of help. Unfortunately the research team was composed only by clinicians without executive capabilities.

The pragmatic nature of our study is an important strength. We have evaluated the effectiveness under real-world conditions in a large section of the population. We included all PCU physicians regardless of their attendance to the training activities, and despite the frequent changes in family physicians and certain organizational changes (creation of new posts, as described in the results). Nevertheless, these facts could dilute the effect of the intervention and could be responsible for the small magnitude of the observed effect.

## Conclusions

The intervention has shown a modest effect on some process variables and on prescribing, with no effect on clinical outcomes in patients. The largest effect has been the increase in CR assessment as a decision tool, a type of assessment that is useful for treatment and management planning. More research is needed to generate evidence on the effectiveness of guideline implementation strategies and to better understand change processes.

Educational interventions focused solely on clinicians seem insufficient to achieve changes in clinical variables. It is likely that interventions at different levels (for professionals, both primary care and specialist), and for patients, related to new forms of health organization, would be able to overcome other barriers such as therapeutic inertia, attitudes of patients and professionals, or poor coordination in health care.

## Additional files

Additional file 1: Clues: Study interventions. Data containing a detailed description of the intervention in control and intervention groups. (DOCX 15 kb)

Additional file 2: Clues: Staff attendance at interventions in primary care units. Data containing a detailed description of staff attendance at interventions in primary care units in both groups (intervention and control). (DOCX 23 kb)

## Abbreviations

ARB: Angiotensin II receptor blockers
CPG: Clinical practice guideline
CR: Coronary risk
FP: Family physician
HbA1c: Glycosylated hemoglobin
ICC: Intracluster correlation coefficient
PCU: Primary care unit
sd: Standard deviation
WMD: Weighted mean difference
