# Supplementary material for: Results from the CLUES study: a cluster randomized trial for the evaluation of cardiovascular guideline implementation in primary care in Spain
Source: BMC Health Serv Res. 2018 Feb 8;18:93. doi: 10.1186/s12913-018-2863-x (PMC5806349; doi:10.1186/s12913-018-2863-x)
Supplement: Supplementary file 1 — Clues: Study interventions. Data containing a detailed description of the intervention in control and intervention groups. (DOCX 15 kb) [file 12913_2018_2863_MOESM1_ESM.docx]

**Clues: Study interventions:**

| **Interventions** | **Specific barriers to be elicited** | **Control group** | **Intervention group** |
| --- | --- | --- | --- |
| Distribution of   printed educational material  Website | Lack of awareness of existing local guidelines;  Lack of availability;  Long formats, usability problems, guideline format no user friendly | Printed guidelines (full guideline and quick reference guide) distributed to physicians and posted on the intranet. | Printed guidelines (full guideline and quick reference guide) distributed to physicians and posted on the intranet.  Specifically designed website with action-oriented recommendations, patient materials, tools (CR calculator, drug formulary…), Q&A site, updates bulletins. |
| Clinical meetings | Passive presentation of guidelines (not interactive), credibility of speakers, acceptance of guidelines. | Local physicians presenting the guidelines in every PCU (trained by guideline authors, "cascade training"). | Guideline authors presenting the guidelines in every PCU  Content and format identical to that of the control group. |
| Workshops with interactive methodology | Understanding, acceptance, passive presentation (not interactive), credibility of trainers, lack of involvement of professionals | ---- | 4-hour workshops based on case studies, delivered by local leaders and focused on the different guideline users. Nurses: CR, diabetic foot.  Family physicians: CR. |

PCU: primary health unit. CR: coronary risk
