# Supplementary material for: Results from the CLUES study: a cluster randomized trial for the evaluation of cardiovascular guideline implementation in primary care in Spain
Source: BMC Health Serv Res. 2018 Feb 8;18:93. doi: 10.1186/s12913-018-2863-x (PMC5806349; doi:10.1186/s12913-018-2863-x)
Supplement: Supplementary file 2 — Clues: Staff attendance at interventions in primary care units. Data containing a detailed description of staff attendance at interventions in primary care units in both groups (intervention and control). (DOCX 23 kb) [file 12913_2018_2863_MOESM2_ESM.docx]

**^Clues: Staff attendance at interventions in primary care units^**

^Medical and nursing staff attendance at interventions in primary care units: intervention group^

| ^Primary Care Units^ | ^Clinical meetings^ | | ^Coronary risk workshops^ | | ^Diabetic foot workshops^ | ^Number of visits to the website^ |
| --- | --- | --- | --- | --- | --- | --- |
|  | ^Medical staff (%)^ | ^Nursing staff (%)^ | ^Medical staff (%)^ | ^Nursing staff (%)^ | ^Nursing staff^  ^(%)^ | ^Number of visits per physician^ |
| ^Amara Berri^ | ^71.4^ | ^50.0^ | ^50.0^ | ^53.3^ | ^73.3^ | ^121^ |
| ^Amara Centro^ | ^90.0^ | ^40.0^ | ^40.0^ | ^70.0^ | ^80.0^ | ^76^ |
| ^Egia^ | ^62.5^ | ^62.5^ | ^62.5^ | ^55.6^ | ^77.8^ | ^87^ |
| ^Hernani-Urnieta-Astigarraga^ | ^68.7^ | ^62.5^ | ^62.5^ | ^75.0^ | ^87.5^ | ^237^ |
| ^Hondarribi^ | ^75.0^ | ^75.0^ | ^75.0^ | ^100.0^ | ^71.4^ | ^127^ |
| ^Intxaurrondo^ | ^71.4^ | ^57.1^ | ^57.1^ | ^100.0^ | ^57.1^ | ^151^ |
| ^Iztieta^ | ^84.6^ | ^53.8^ | ^53.8^ | ^66.7^ | ^53.3^ | ^205^ |
| ^Loiola^ | ^100.0^ | ^50.0^ | ^50.0^ | ^50.0^ | ^75.0^ | ^239^ |
| ^Ondarreta^ | ^57.1^ | ^14.3^ | ^14.3^ | ^56.2^ | ^56.2^ | ^76^ |
| ^Pasai San Pedro^ | ^66.7^ | ^66.7^ | ^66.7^ | ^66.7^ | ^83.3^ | ^529^ |
| ^Zarautz-Orio- Aia^ | ^81.2^ | ^68.7^ | ^68.7^ | ^62.5^ | ^62.5^ | ^209^ |
| ^Bombero Etxaniz^ | ^47.4^ | ^68.4^ | ^68.4^ | ^90.0^ | ^40.0^ | ^22^ |
| ^Basurto-Altamira^ | ^84.6^ | ^100.0^ | ^100.0^ | ^100.0^ | ^23.1^ | ^38^ |
| ^Casco Viejo^ | ^71.4^ | ^100.0^ | ^100.0^ | ^100.0^ | ^14.3^ | ^95^ |
| ^Gazteleku^ | ^57.1^ | ^85.7^ | ^85.7^ | ^100.0^ | ^53.3^ | ^110^ |
| ^Indautxu^ | ^83.3^ | ^83.3^ | ^83.3^ | ^100.0^ | ^21.4^ | ^6^ |
| ^La Merced^ | ^100.0^ | ^100.0^ | ^100.0^ | ^100.0^ | ^50.0^ | ^81^ |
| ^Rekalde^ | ^52.9^ | ^94.1^ | ^94.1^ | ^100.0^ | ^20.0^ | ^100^ |
| ^San Adrian^ | ^88.9^ | ^100.0^ | ^100.0^ | ^100.0^ | ^42.9^ | ^48^ |
| ^San Ignacio^ | ^46.7^ | ^80.0^ | ^80.0^ | ^0.0^ | ^46.7^ | ^21^ |
| ^Zorroza-Alonsotegui^ | ^70.0^ | ^90.0^ | ^90.0^ | ^50.0^ | ^70.0^ | ^0 (*)^ |
| **^Total^** | **^69.6%^** | **^71.3%^** | **^71.3%^** | **^75.0%^** | **^52.8%^** | **^109^** |

^(*) For technical reasons, it was not possible to activate the Website in Zorroza-Alonsotegui.^

^Medical and nursing staff attendance at interventions in primary care units: control group^

| ^Primary Care Units^ | **^Clinical meetings^** | |
| --- | --- | --- |
|  | **^% Physician staff^** | **^% Nursing staff^** |
| ^Parte Vieja^ | ^42.9^ | ^25.0^ |
| ^Alza^ | ^50.0^ | ^66.7^ |
| ^Beraun^ | ^80.0^ | ^72.7^ |
| ^Bidebieta^ | ^60.0^ | ^100.0^ |
| ^Dunboa^ | ^75.0^ | ^86.7^ |
| ^Gros^ | ^100.0^ | ^88.2^ |
| ^Irun^ | ^27.8^ | ^36.8^ |
| ^Lasarte-Usurbil^ | ^69.2^ | ^53.8^ |
| ^Lezo-Pasajes San Juan^ | ^66.7^ | ^100.0^ |
| ^Oiartzun^ | ^66.7^ | ^60.0^ |
| ^Pasajes Antxo^ | ^75.0^ | ^75.0^ |
| ^Zumaia-Zestoa-Getaria^ | ^77.8^ | ^37.5^ |
| ^Begoña^ | ^100.0^ | ^80.0^ |
| ^Bolueta^ | ^62.5^ | ^37.5^ |
| ^La Peña^ | ^100.0^ | ^71.4^ |
| ^Deusto^ | ^56.3^ | ^66.7^ |
| ^Karmelo^ | ^66.7^ | ^60.0^ |
| ^Javier Saenz de Buruaga^ | ^37.5^ | ^75.0^ |
| ^Otxarkoaga^ | ^100.0^ | ^45.5^ |
| ^Santutxu-Solokoetxe^ | ^66.7^ | ^42.9^ |
| ^Txurdinaga^ | ^44.4^ | ^70.0^ |
| ^Zurbaran^ | ^75.0^ | ^71.4^ |
| **^Total^** | **^66.7%^** | **^62.7%^** |
